# Risk of Second Cancer in Hodgkin Lymphoma Survivors and Influence of Family History

**DOI:** 10.1200/JCO.2016.70.9709

**Published:** 2017-03-13

**Authors:** Amit Sud, Hauke Thomsen, Kristina Sundquist, Richard S. Houlston, Kari Hemminki

**Affiliations:** Amit Sud and Richard S. Houlston, The Institute of Cancer Research, London, United Kingdom; Hauke Thomsen and Kari Hemminki, German Cancer Research Centre, Heidelberg, Germany; and Kristina Sundquist and Kari Hemminki, Centre for Primary Health Care Research, Lund University, Malmö, Sweden.

## Abstract

**Purpose:**

Although advances in Hodgkin lymphoma (HL) treatment have led to improved disease-free survival, this has been accompanied by an increased risk of second cancers. We sought to quantify the second cancer risks and to investigate the impact of family history.

**Patients and Methods:**

Using the Swedish Family-Cancer Project Database, we identified 9,522 individuals with primary HL diagnosed between 1965 and 2012. We calculated standardized incidence ratios and cumulative incidence of second cancer in HL survivors and compared the standardized incidence ratios of lung, breast, colorectal, and all second cancers in HL survivors with and without a site-specific family history of cancer. Interactions between family history of cancer and HL treatment were evaluated under additive and multiplicative models.

**Results:**

Overall, the risk of a second cancer in HL survivors was increased 2.39-fold (95% CI, 2.29 to 2.53). The 30-year cumulative incidence of breast cancer in women diagnosed with HL at younger than 35 years of age was 13.8%. We observed no significant difference in cancer risk over successive time periods. The risk of all second cancers was 1.3-fold higher for HL survivors with a first-degree relative with cancer (*P* < .001), with 3.3-fold, 2.1-fold, and 1.8-fold differences shown for lung, colorectal, and breast cancers, respectively. Moreover, a greater than additive interaction between family history of lung cancer and HL treatment was shown (*P* = .03).

**Conclusion:**

HL survivorship is associated with a substantive risk of a second cancer. Notably, the risk is higher in individuals with a family history of cancer. This information should be used to inform risk-adapted therapy and to assist in screening to reduce long-term morbidity and mortality in patients with HL.

## INTRODUCTION

Advances in the management of Hodgkin lymphoma (HL) over the past 40 years have led to improved disease-free survival in patients.^1^ However, this comes at the cost of an increased risk of second cancers, cardiovascular disease, and other treatment-related complications.^2-13^ The risk of second cancers in HL survivors, which persists for many decades after treatment, has been reported to be influenced by various factors, including age at treatment,^3^ site and dose of radiotherapy,^14^ chemotherapy,^5^ and smoking.^15^ The use of treatment regimens based on a reduction in the field and dose of radiotherapy and alkylating chemotherapy has been introduced to reduce rates of long-term complications while maintaining a high cure rate.^1^ Despite such modifications, a recent study from the Netherlands showed that this has not affected the risk of second cancers in patients with HL^12^.

A family history of breast cancer was first suggested to be a risk factor for second cancer in HL nearly 20 years ago,^16^ and it has long been postulated that a subset of patients with cancer display a high sensitivity to mutational agents because of a genetic predisposition.^17^ Evidence for such an assertion in the context of HL is provided by an analysis of a Swedish cohort of patients with HL, but the power of the study did not allow the impact of family history to be studied in detail.^18^ Moreover, the analysis included HL survivors with a prior history of cancer other than HL, which potentially biased the conclusions.

To gain insight into the risk of second cancer after a diagnosis of HL and its relationship to temporal changes in treatment regimens, we analyzed data on a cohort of 9,522 Swedish patients. In addition, through the use of the Swedish Family-Cancer Project Database, we performed an updated analysis of the influence of family history, as a surrogate for genetic susceptibility, on the risk of second cancer in patients with HL.

## PATIENTS AND METHODS

### Patients

The Swedish Family-Cancer Project Database was created by linking information from the multigeneration register, national censuses, the Swedish Cancer Registry, and death notifications.^19^ The Swedish Cancer Registry, established in 1958, is based on the compulsory reporting of all diagnosed patients, thereby providing near-complete coverage of all cancer registrations in Sweden.^20^ There is an under-representation of individuals in the first generation in some families; however, this has not been shown to adversely bias estimates of familial risk.^21,22^ In this study, we analyzed all incident cases of HL between 1965 and 2012. Individuals with a diagnosis of malignancy before HL were excluded. Individuals with HL were observed until December 31, 2012, time of migration from Sweden, or death. Data regarding HL histologic subtype were available for all individuals diagnosed since 1993. The database includes the date and site of occurrence of up to four subsequent new malignancies after diagnosis of HL and dates and causes of death. Cancers in the first year after HL diagnosis were omitted from our analysis because of the likelihood of excess cases as a result of increased surveillance.^23^ First-degree relatives (FDRs) of individuals with HL, as well as the dates and sites of cancer diagnoses in the FDRs, were identified. The study was undertaken with approval from the ethics committee at Lund University, Sweden, and was conducted in accordance with the tenets of the Declaration of Helsinki.

### Statistical Analysis

Expected numbers of cancers were computed using 5-year age, sex, and calendar period incidence rates for Sweden. Observed numbers were compared with expected numbers by means of the standardized incidence ratio (SIR) assuming a Poisson distribution. The risk of a second malignancy was estimated for different time intervals after treatment of HL. The absolute excess risk (AER) was calculated as the observed number of second cancers in our cohort minus that expected, divided by the number of person-years at risk, multiplied by 10,000. SIRs were calculated in the HL cohort and were stratified by patient characteristics. Tests for trend in SIRs were performed by evaluating the likelihood function in collapsed person-time additive Poisson regression models with and without the inclusion of the variable. Patients in whom multiple second cancers were diagnosed were counted only once in the analysis of all second cancers; in this analysis, the time at risk ended on the date on which a second cancer was diagnosed. For the site-specific cancer analyses, the time at risk ended on the date on which the site-specific cancer was diagnosed, regardless of whether this was preceded by another cancer. The cumulative incidence of second cancer was estimated with death treated as a competing risk.^24^ Interaction contrast ratios (ICRs) and multiplicative interaction indexes (MIIs) were used to investigate the possible interaction between HL treatment and family history of cancer:^25^ ICR = SIR_cancerxfh_ − SIR_cancer_ − SIR_fh_ + 1 and MII = SIR_cancerxfh_/(SIR_cancer_ × SIR_fh_), where SIR_cancer_ is the relative risk (RR) of cancer in HL survivors, SIR_fh_ is the RR associated with having an affected FDR, and SIR_cancerxfh_ is the RR of cancer in HL survivors having an affected FDR. MII > 1 signifies greater than multiplicative interaction, and ICR > 0 signifies a positive interaction or more than additivity. The relative survival rate was calculated as the ratio of the observed survival rate to the expected survival rate in Sweden, matched by age, sex, and calendar year.^26,27^ Statistical analyses were performed using Stata version 14 (STATA, College State, TX) and R version 3.3.1 software.^28^ A *P* value ≤ .05 (two-sided) was considered statistically significant, although we acknowledge we have presented the results of many statistical tests, and therefore caution against the overinterpretation of our findings, especially when they are based on *P* values > .001.

## RESULTS

### Patients and Record Linkage

From the Swedish Family-Cancer Project Database, data on 9,522 patients with a primary diagnosis of HL between 1965 and 2013 were analyzed. Of the 9,522 patients 5,488 were male and 4,034 were female, with a mean age at diagnosis of 49 years (Table 1). Five thousand seven hundred twenty-one were deceased and 129 had emigrated before the end of the study period. The median follow-up was 12.6 years, with the longest being 48 years. Of patients with HL in whom tumor with histology had been recorded, 1,839 (54%) were nodular sclerosis HL (NSHL) and 539 (16%) were of mixed cellularity HL. Among the 3,917 individuals who died > 1 year after the diagnosis of HL, 842 (9%) died with the occurrence of a subsequent cancer during the follow-up.

**Table 1.**
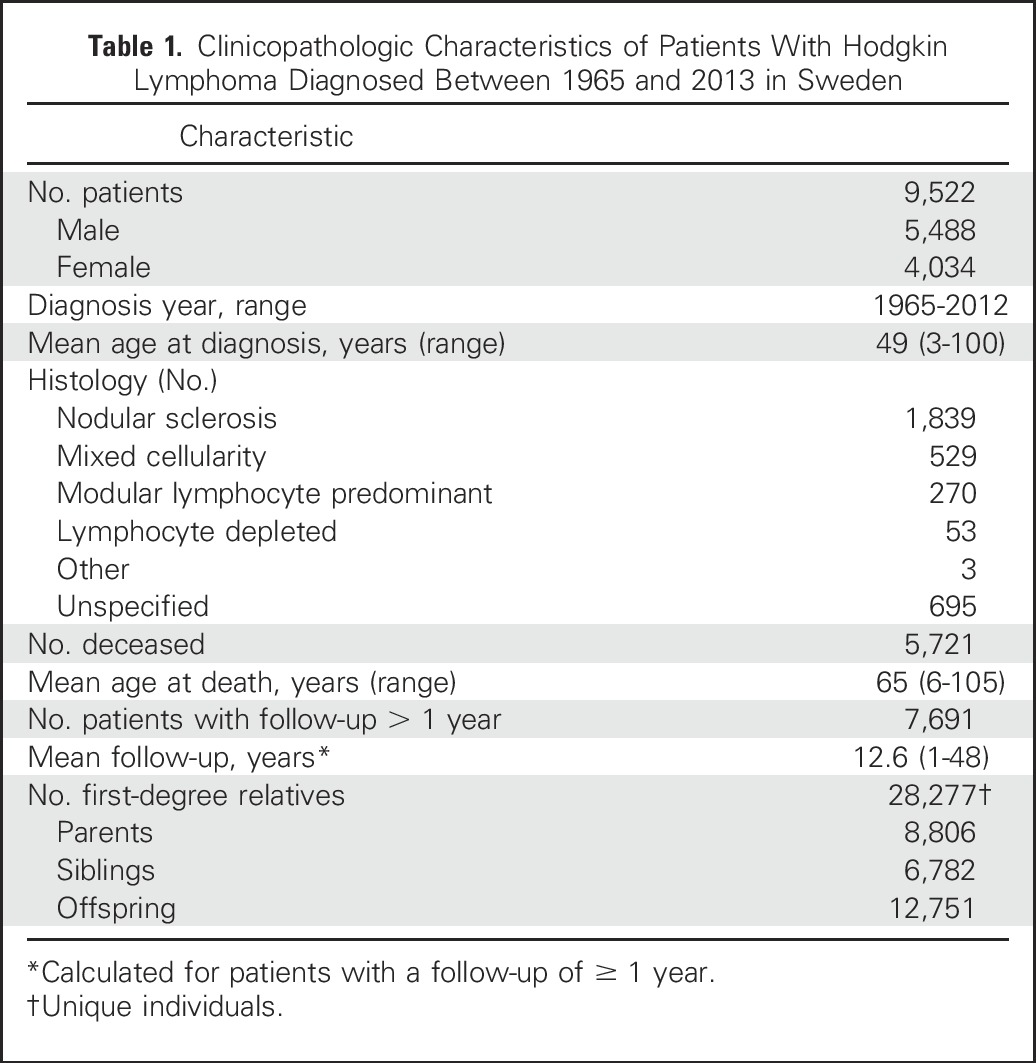
Clinicopathologic Characteristics of Patients With Hodgkin Lymphoma Diagnosed Between 1965 and 2013 in Sweden

### Risk of Second Cancer in Patients With HL

A total of 1,215 second cancers were observed in 1,121 patients (12% of patients with HL). The risk of all second cancers was elevated significantly after HL diagnosis, with a SIR of 2.39 (95% CI, 2.25 to 2.53), translating to an AER of 71.2 cases per 10,000 person-years (Table 2). In the nonstratified analysis, non-Hodgkin lymphoma (NHL) contributed the most to the AER (16.2% of the excess cancer risk), followed by lung cancer (14.5% of the excess cancer risk), breast cancer (12.9% of the excess cancer risk), nonmelanoma skin cancers (11.4% of the excess cancer risk), leukemia (9.7% of the excess cancer risk), and colorectal cancer (7.6% of the excess cancer risk). The SIR for all second cancers remained high > 30 years after treatment of HL, although the patterns of excess risk observed at the different intervals differed depending on the cancer site (Table 2).

**Table 2.**
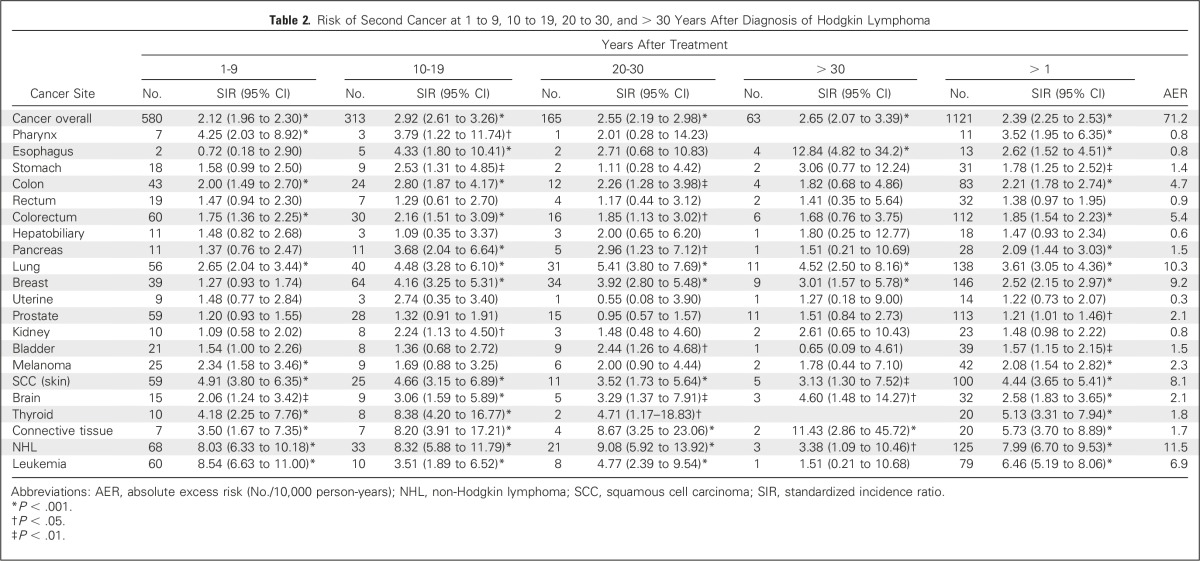
Risk of Second Cancer at 1 to 9, 10 to 19, 20 to 30, and > 30 Years After Diagnosis of Hodgkin Lymphoma

### Influence of Sex, Age, and Tumor Subtype on Second Cancer Risk

**Fig 1.**
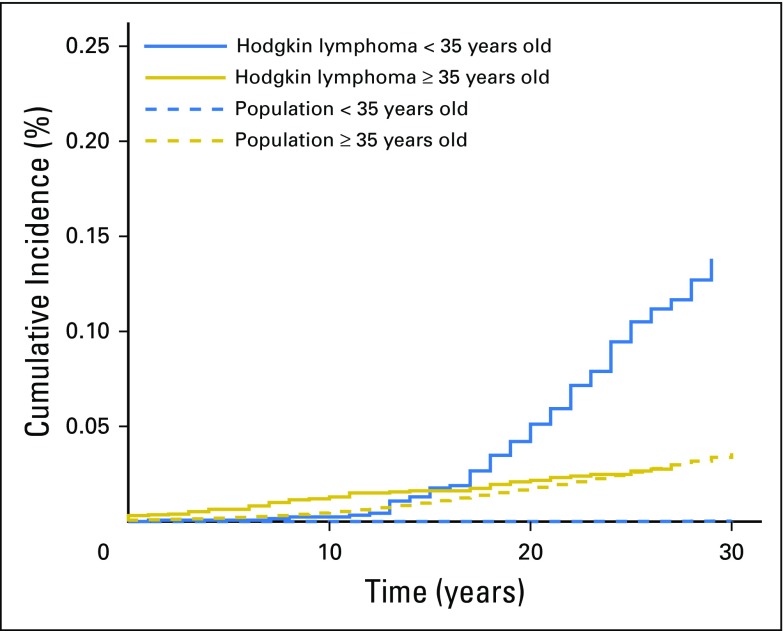
Cumulative incidence of breast cancer in female survivors of Hodgkin lymphoma, by age at Hodgkin lymphoma diagnosis, with death treated as a competing risk. The solid blue line represents women diagnosed with Hodgkin lymphoma at younger than 35 years of age, and the solid gold line represents women diagnosed with Hodgkin lymphoma at 35 years of age or older. The dashed blue line represents women in the population younger than 35 years of age, and the dashed gold line represents women in the population 35 years of age or older.

For men diagnosed with HL before the age of 35 years, the SIRs for all second cancers, colorectal cancer, lung cancer, NHL, and leukemia were higher when compared with those diagnosed with HL after the age of 35 years. Similarly, for women diagnosed with HL before the age of 35 years, the SIRs for all second cancers, breast cancer, lung cancer, and NHL were higher when compared with women diagnosed with HL after the age of 35 years (Table 3). In women diagnosed with HL at younger than 35 years of age, the 30-year cumulative risk of breast cancer was 13.8% (95% CI, 11.1 to 16.9), which accounted for > 50% of the AER in this age group of women. This contrasted with women diagnosed with HL at older than 35 years of age for whom the 30-year cumulative incidence of breast cancer was only 3.3% (95% CI, 2.2 to 3.9), and accounted for < 3% of the AER (Fig 1 and Data Supplement). Given the difference in cause and tumor biology of HL histologic subtypes, we investigated whether second cancer risk might also differ. We observed similar SIRs for cancer overall and the common site-specific cancers for the most common subtype, NSHL (Data Supplement). We do acknowledge, however, that this observation should be interpreted with caution because numbers are small and the unclassified HL cases include a proportion of NSHL.

**Table 3.**
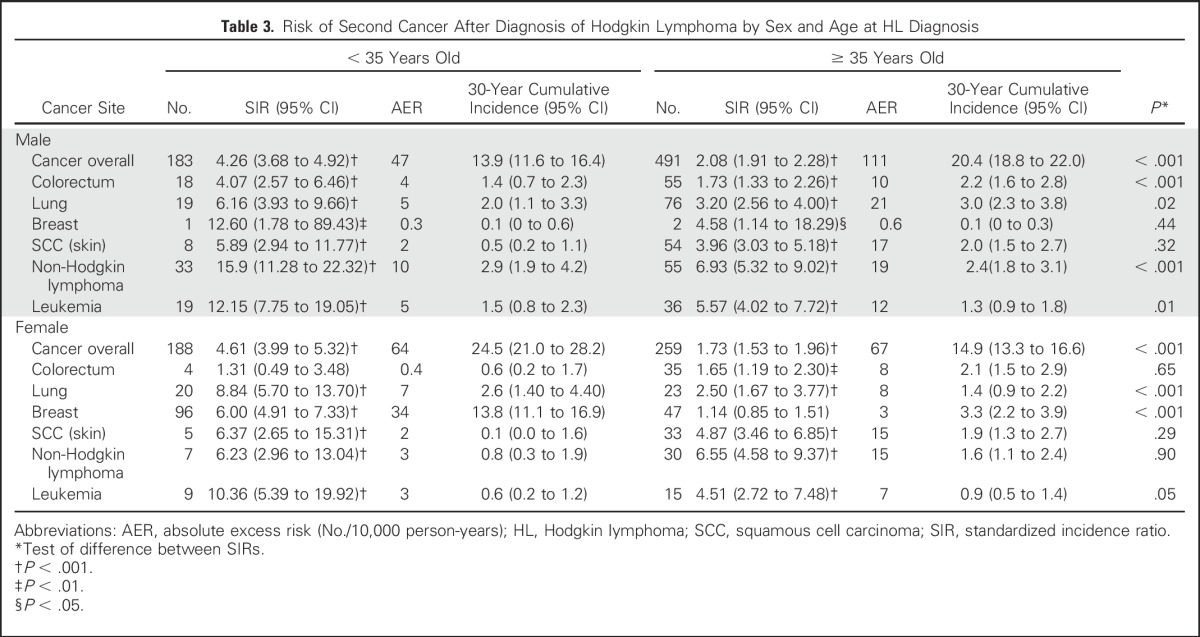
Risk of Second Cancer After Diagnosis of Hodgkin Lymphoma by Sex and Age at HL Diagnosis

### Temporal Effects on Risk of Second Cancer

Although details of individual patient therapy are not registered by the Swedish Cancer Registry, the treatment principles for HL in Sweden are broadly similar to those of other Western countries.^29^ Briefly, extended field irradiation, mainly mantle field, was the standard treatment of patients with HL during the early phase of our analysis. Patients received radiotherapy, chemotherapy, or a combined modality treatment. For those patients treated after 1990, less toxic regimens were being introduced.^29,30^ Partitioning data, we analyzed the second cancer risk for patients diagnosed with HL in the time periods of 1965 to 1977, 1978 to 1988, and 1989 to 2000. We found little evidence of a change in SIRs for overall cancer and for the most common sites of cancer, including hematopoietic malignancy (Data Supplement).

### Impact of Family History on Risk of Second Cancer and Survival

From the Multigenerational Register, a total of 28,277 FDRs of the 9,522 patients with HL were identified. The SIR for cancer risk in FDRs was 1.02 (95% CI, 0.99 to 1.06). In the HL survivors, 2,785 individuals (29%) had one or more FDRs with a family history of cancer (Data Supplement). We found an increase in second cancer risk in HL survivors who had an FDR with cancer, when compared with HL survivors with no FDR with cancer (*P* < .001) with SIRs of 2.83 (95% CI, 2.58 to 3.10) and 2.16 (95% CI, 2.00 to 2.33), respectively. Moreover, the increased risk of second cancer was correlated with the number of FDRs affected with cancer, respective SIRs being 2.67 (95% CI, 2.40 to 2.97) and 3.40 (95% CI, 2.85 to 4.09) for patients with one and two or more affected FDRs (*P* < .001; Data Supplement).

In an analysis of colorectal, breast, and lung cancer, we observed elevated risks of second cancers in HL survivors with an FDR with the corresponding site-specific cancer. For lung cancer, SIRs were 11.24 (FDR with lung cancer; 95% CI, 6.38 to 19.79) and 3.39 (no FDR with lung cancer; 95% CI, 2.85 to 4.03; *P* < .001). For colorectal cancer, SIRs were 3.71 (FDR with colorectal cancer; 95% CI, 2.05 to 6.70) and 1.76 (no FDR with colorectal cancer; 95% CI, 1.45 to 2.14; *P* = .03). Finally, for breast cancer, SIRs were 4.36 (FDR with breast cancer; 95% CI, 2.60 to 6.55) and 2.36 (no FDR with breast cancer; 95% CI, 1.98 to 2.81; *P* = .04; Table 4). Moreover, for lung cancer, a more than additive interaction between family history and HL treatment was shown (*P* = .03; Table 5). Overall, having a family history of cancer was shown not to influence survival from a second cancer in patients with HL (Data Supplement).

**Table 4.**
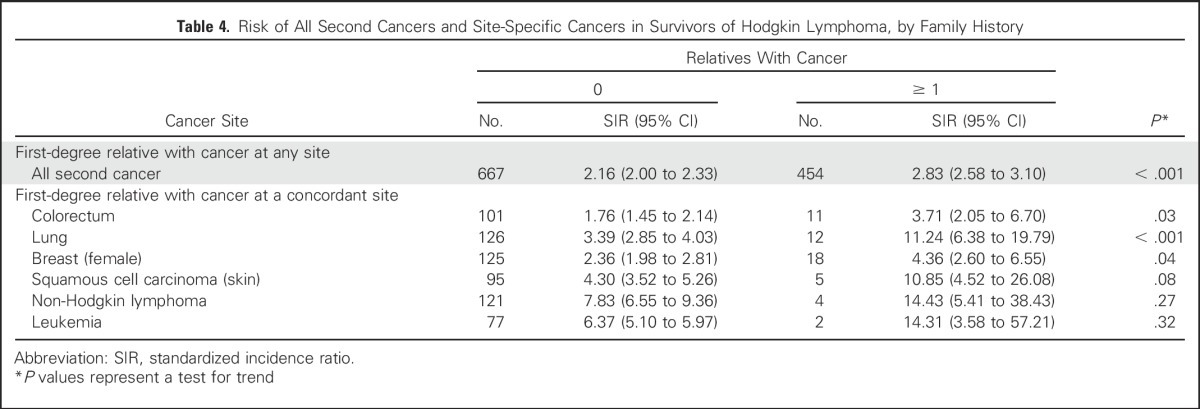
Risk of All Second Cancers and Site-Specific Cancers in Survivors of Hodgkin Lymphoma, by Family History

**Table 5.**
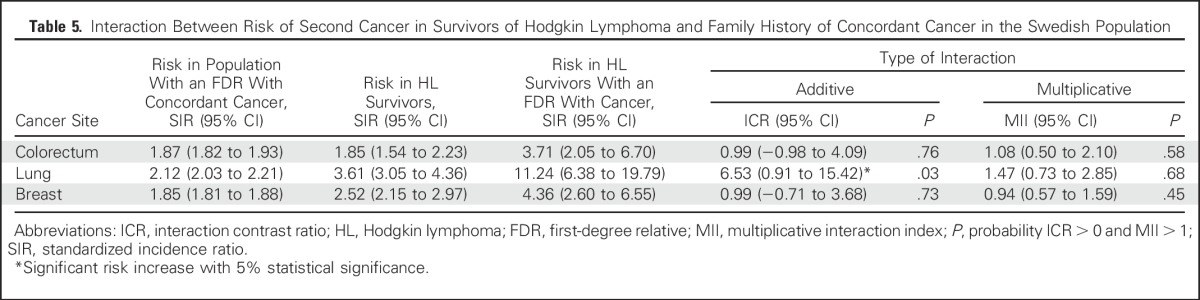
Interaction Between Risk of Second Cancer in Survivors of Hodgkin Lymphoma and Family History of Concordant Cancer in the Swedish Population

## DISCUSSION

This analysis provides further evidence that survivorship from HL is associated with a significant risk of a second cancer. Furthermore, we confirm the previous findings of a relationship between age at diagnosis and sex, and the risk of second cancers. Our analysis also shows that differences in patient management over successive decades have not led to a lessening of risk of second cancers in HL survivors, an observation consistent with recent data from the Netherlands.^12^ Possible reasons for this observation include the impact of screening or that the risk is maintained because of an interaction between less toxic chemotherapy and second cancer risk. For example, higher doses of alkylating agents, which are more likely to cause premature menopause, have been reported to reduce radiation-induced breast cancer risk.^31^ In addition, although patients diagnosed with HL are likely to have received lower doses of alkylating agents in recent years, the omission of radiotherapy from such treatment regimens is likely to have led to an increased proportion of all patients receiving alkylating agents.^12^ Finally, we cannot exclude entirely the possibility that the study periods we analyzed were too short or early to be able to demonstrate a difference.

In this study, HL survivors with a family history of colorectal, lung, or breast cancer showed an increased risk of concordant second cancers when compared with HL survivors without a family history. To our knowledge, this is the first population study to demonstrate site-specific second cancer risk after HL being influenced by family history. Our findings support the notion of familial determinants of second cancer risk being consistent with inherited genetic predisposition. Direct evidence for such a model is provided by the example of retinoblastoma, in which individuals with hereditary retinoblastoma have a much higher risk of radiotherapy-induced second malignancy compared with those with sporadic disease.^32,33^ Thus far, no similar high-impact mutation has been identified for HL, and sequencing of *TP53*, *BRCA1*, *BRCA2*, and *ATM* in HL survivors with a second malignancy has not substantiated a role for mutations in these genes as a cause of subsequent cancer risk.^16,34^ Polygenic susceptibility provides an alternative explanation for genetic susceptibility, whereby the elevated risk is enshrined in common genetic variants, which, in isolation, exert small effects but act in concert to have a relatively profound impact. Such an assertion is supported by the recent findings that variation in *FGFR2* and *PRDM1* influences the risk of second cancer in HL survivors.^35,36^ For lung cancer, we were able to demonstrate a greater than additive interaction between family history of lung cancer and HL treatment, which may be the consequence of additional shared nongenetic risk factors, most probably a propensity to smoke. This interaction is of significant importance because it may explain, in part, why no significant change in second lung cancer has been observed despite modifications in treatment regimens. The number of cases of breast and colorectal cancer did not permit the rejection of any interaction model.

Survival of patients with HL after diagnosis of a second cancer was not significantly worse in those with an affected FDR. Moreover, noninferiority of survival in individuals with a family history of cancer has been described in colorectal, breast, and prostate cancer.^37-39^ Although it remains to be established, reasons for this may include increased cancer surveillance, resulting in presentation at earlier stages of disease,^40,41^ improved health-related behavior,^42^ or differences in tumor biology and response to therapy.

Our study has major strengths. First, we have avoided ascertainment bias in patient selection because our cohort analysis was based on the Swedish population, for which there is near complete case registration with long-term follow-up.^20^ Second, the Swedish Family-Cancer Project Database includes a large number of individuals linked to family members, and this has allowed us to uniquely study the influence of family history on cancer risk.^19^ We do acknowledge, however, that a limitation of our study is the reliance on year of treatment as an effective surrogate for type of treatment and that we did not have the opportunity to incorporate information on risk factors such as smoking. However, most of the familial risk of lung cancer is a result of a predisposition to smoke. Hence, by performing an interactive analysis, we addressed this area.

Despite these caveats, our findings further substantiate the significant cancer risks associated with survivorship from HL and that these are modified by a family history of cancer. In addition, our findings are of importance in a primary health care setting where many surviving patients with HL are treated (for other symptoms and diseases) after the first 5 years of follow-up. Long-term understanding of the biologic basis of these associations offers the prospect of personalizing therapy in HL. However, such information has current value clinically, when planning risk-adapted therapy for patients with HL. Furthermore, our findings with respect to lung cancer emphasize the importance of instigating programs to reduce smoking in patients with HL. Finally, as well as offering breast cancer screening to women who have received supradiaphragmatic radiotherapy,^43,44^ obtaining family history information has a place in informing the long-term follow-up screening of all patients with HL.
